# Acquired Focal Choroidal Excavation Secondary to Pachychoroid Choroidal Neovascular Membrane After Central Serous Chorioretinopathy

**DOI:** 10.1177/24741264231163395

**Published:** 2023-04-10

**Authors:** Anubhav Garg, Husam Khaleel, Charbel Wahab, Peng Yan

**Affiliations:** 1 Department of Surgery, Division of Ophthalmology, McMaster University, Hamilton, ON, Canada; 2 Department of Ophthalmology and Vision Sciences, University of Toronto, Toronto, ON, Canada; 3 Kensington Vision and Research Centre, Toronto, ON, Canada

**Keywords:** acquired, central serous chorioretinopathy, choroidal neovascular membrane, focal choroidal excavation, pachychoroid spectrum

## Abstract

**Purpose:** To report a case of acquired conforming-type focal choroidal excavation (FCE) secondary to a pachychoroid choroidal neovascular membrane (CNVM) triggered by central serous chorioretinopathy (CSCR). **Methods:** A case and its findings were analyzed. **Results:** A 54-year-old Asian man who had spontaneous resolution of CSCR in the right eye presented with a pachychoroid CNVM and FCE 1 year after the initial CSCR diagnosis. Intravitreal antivascular endothelial growth factor injections were initiated, and the subretinal fluid and intraretinal hemorrhage resolved. The patient was followed for FCE progression for 3 years. **Conclusions:** Acquired FCE can occur secondary to CSCR and pachychoroid CNVM. The pathogenesis may be focal choroidal ischemia, choroidal vascular collapse, and fibrosis leading to choroidal excavation. This case highlights the progression of the spectrum of pachychoroid disorders from CSCR, pachychoroid CNVM, and subsequent acquired confirming-type FCE. Further research is needed to assess other diseases leading to acquired FCE and to determine the underlying mechanism.

## Introduction

Focal choroidal excavation (FCE) is a concavity in the choroid detected by optical coherence tomography (OCT). It was originally described by Jampol et al^1^ and named by Margolis et al.^2^

Although FCE has classically been considered to be congenital,^2^ there have been reports of acquired FCE after choroidal neovascular membrane (CNVM) formation^3,4^ and multiple evanescent white-dot syndrome (MEWDS).^5^ This has led to the pathogenesis and prognosis of FCE being unclear. A recent review by Verma et al^6^ proposed an etiology-based classification in which FCE is characterized as congenital/primary (ie, no associated evidence of other chorioretinal disease, generally stable, and does not threaten vision) or acquired/secondary (ie, acquired after birth, may progress and threaten vision).

Although FCE has been associated with pachychoroid-spectrum diseases including central serous chorioretinopathy (CSCR),^7^ to our knowledge there are no reports documenting acquired FCE secondary to pachychoroid CNVM specifically occurring after CSCR. Here, we present a case of acquired FCE occurring after a pachychoroid CNVM developed in a patient who had CSCR that spontaneously resolved.

## Case Report

**Figure 1.**
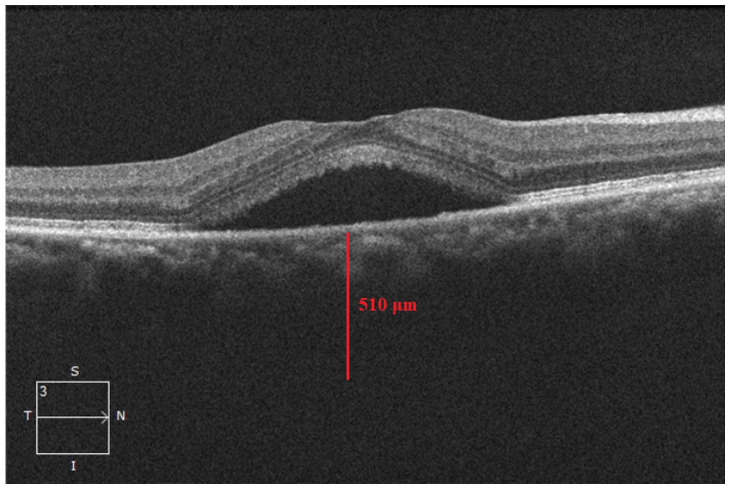
Subretinal fluid consistent with central serous chorioretinopathy in the right eye. The subfoveal choroidal thickness is 510 μm.

A 54-year-old Asian man with no ocular or medical history was diagnosed with CSCR in the right eye in June 2018 (Figure 1). The visual acuity (VA) in that eye was 20/30. The CSCR spontaneously resolved 1 month later with a VA of 20/20. The patient was stable with a VA of 20/20 at the 5-month follow-up.

**Figure 2.**
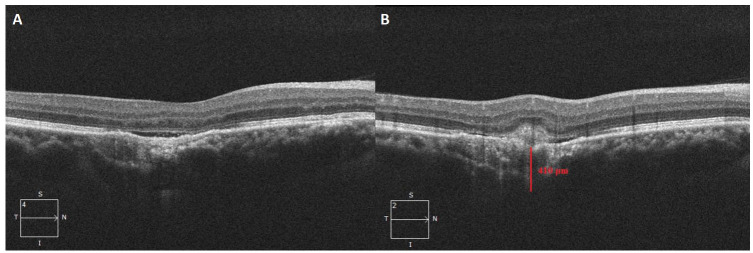
(A) Focal choroidal excavation with thickened choroid and (B) subretinal hyperreflective material with subretinal fluid 1 year after the initial diagnosis of central serous chorioretinopathy. The choroidal thickness is 410 μm at the choroidal neovascular membrane.

One year after his CSCR diagnosis, the patient presented with metamorphopsia and reduced VA of 20/20^−2^ OD. An examination and imaging showed a new onset of subretinal fluid and intraretinal hemorrhage with CNVM and FCE (Figure 2). He was immediately started on intravitreal antivascular endothelial growth factor injection treatment, and the subretinal fluid and hemorrhage resolved. He received monthly injections of aflibercept 2 mg/0.05 mL over 6 months. No recurrence of subretinal fluid was noted.

**Figure 3.**
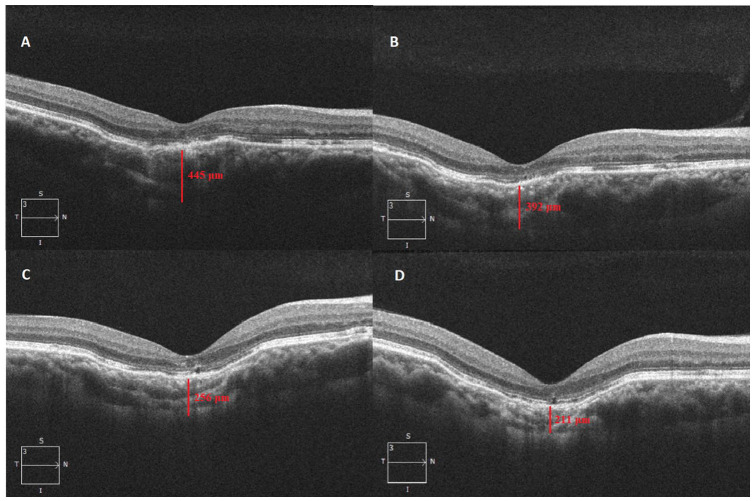
Follow-up optical coherence tomography imaging shows conforming-type focal choroidal excavation (A) 16 months, (B) 21 months, (C) 29 months, and (D) 37 months after the diagnosis of focal choroidal excavation. The corresponding subfoveal choroidal thickness is (A) 445 μm, (B) 392 μm, (C) 256 μm, and (D) 211 μm.

**Figure 4.**
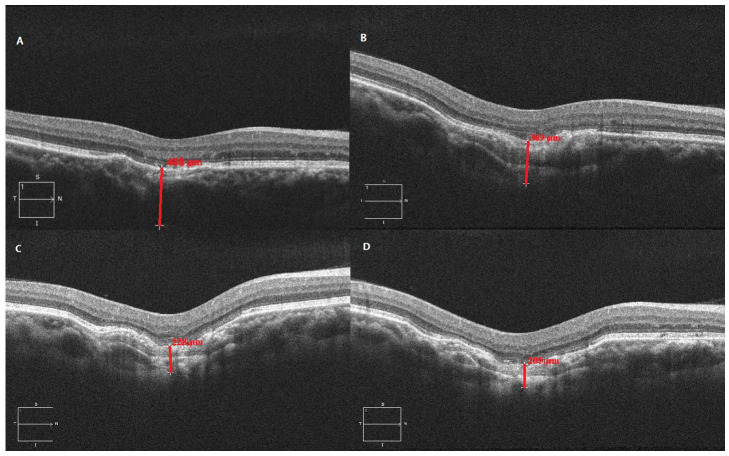
Follow-up optical coherence tomography imaging shows the choroidal thickness at the initial location of the choroidal neovascular membrane at (A) first presentation (488 μm), (B) the 2-year follow-up (369 μm), (C) the 3-year follow-up (228 μm), and (D) the 4-year follow-up (205 μm). (B) The choroidal thickness at the 1-year follow-up was 410 μm.

The patient was followed regularly with serial imaging and measurement of the progression of FCE at the subfovea and at the initial location of the CNVM for 3 more years (Figures 3 and 4). The rate of excavation progression was 74.75 μm per year at the subfovea and 70.75 μm per year at the initial location of the CNVM. The VA in the right eye at the final follow-up was 20/50^−2^. The anterior segment and intraocular pressure in that eye were normal at all appointments. The left eye remained asymptomatic but with the presence of pachychoroid. The VA in that eye was 20/20, with an unremarkable anterior and posterior segment examination and normal imaging at all appointments.

## Conclusions

Although FCE was originally considered to be a congenital lesion,^2^ recent literature has reported acquired FCE after CNVM^3,4^ and MEWDS,^5^ leading to continued discussion regarding the pathogenesis of FCE. To our knowledge, our case is the first to demonstrate that conforming-type FCE can occur specifically after the progression of CSCR to pachychoroid CNVM, further supporting acquired subtypes of FCE while documenting a unique pathology in CSCR preceding FCE. In addition, given the association with pathology such as CSCR and pachychoroid CNVM, recent literature has considered FCE to be a possible variation of the pachychoroid spectrum.^6^ Our case further warrants inclusion of FCE in the spectrum of pachychoroid disorders because in this patient with pachyvessels, we observed a progression from CSCR to FCE with pachychoroid CNVM.

A study by Ellabban et al^8^ suggests an association between FCE and CSCR given that FCE developed in 7.8% of 116 eyes with CSCR. However, the authors were uncertain whether the lesions were congenital or acquired. Our case shows that CSCR can trigger progression to pachychoroid CNVM and then FCE formation.

Ellabban et al^8^ also postulated the mechanism by which FCE develops in association with CSCR. Some of their cases showed unusual hyperreflective choroid tissue devoid of large choroidal vessels under the FCE that bridged the outer choroidal boundary with the FCE. They hypothesized that this bridging tissue represented scarring in the choroidal connective tissue, which contracted to form the FCE by retracting the overlying retinal pigment epithelium. We observed a similar hyperreflectivity on OCT, suggestive of focal choroidal ischemia. The collapse of choroidal vessels might have led to the formation of choroidal fibrotic tissue, which subsequently played a role in the development of the FCE. However, given that not all reported cases of FCE are associated with the spectrum of pachychoroid disorders, other mechanisms could contribute to the development of FCE.^8^

In conclusion, FCE can be acquired after pachychoroid spectrum disorders such as CSCR and pachychoroid CNVM. The mechanism of acquired FCE from CSCR and pachychoroid CNVM may be related to focal choroidal ischemia, collapse of choroid vessels, and fibrosis. Furthermore, FCE appears to be associated with the pachychoroid spectrum of diseases. Further research is required to elucidate other pathologies associated with acquired FCE and assess the mechanism of FCE development.
